# Work-related movement and lumbar spine direction: a systematic review of lifting, posture, and pain risk

**DOI:** 10.13075/ijomeh.1896.02828

**Published:** 2026

**Authors:** Changli Liu, Donglin Zhang, Wen Si, Zhiming Li, Shengyang Jiang, Long Yin, Zhongwu Ren

**Affiliations:** 1 The 970th Hospital of the Chinese People's Liberation Army Joint Logistic Support Force, Department of Spine Surgery, Yantai, China; 2 Shandong University Dezhou Hospital, Qilu Hospital, Department of Anesthesiology, Dezhou, China; 3 Shandong University Dezhou Hospital, Qilu Hospital, Department of Spine Surgery, Dezhou, China

**Keywords:** biomechanics, lumbar spine, posture, low back pain, occupational lifting, spinal loading

## Abstract

Low back pain (LBP) is a prevalent musculoskeletal condition of occupational and socioeconomic importance. The main biomechanical exposures related to LBP among workers include repetitive lifting, non-neutral trunk postures, and long-duration static trunk positions. This systematic review aimed to evaluate the role of lumbar spine movement direction during work-related lifting and posture in relation to spinal loading and the risk of LBP. PubMed, Scopus, and Web of Science were searched for articles published in January 2015 – June 2025. Eligible studies included adult workers performing occupational lifting or posture activities and reported direction of lumbar spine movement, biomechanical loading measures, or LBP outcomes. Ten studies met the eligibility criteria, including experimental, modeling, and field-based research. Biomechanical evidence showed that squat lifting reduced shear forces at lower vertebral levels but increased overall musculoskeletal effort, while stoop lifting produced higher shear loads at L5/S1. Lifting with a lordotic spine curvature elevated both compressive and shear forces. Individuals with LBP exhibited altered neuromechanical strategies such as reduced lumbar flexion and increased pelvic compensation. Field studies linked repetitive manual handling and constrained postures to higher LBP prevalence. Asymmetrical movement, inadequate strength, and fatigue also contributed to injury risk. The direction and quality of lumbar spine motion during lifting and other posture-dependent activities is an important modifiable risk factor for LBP. Through environmental design changes and behavioral training programs employees can learn and apply ergonomically suitable movement patterns and postures to prevent LBP and decrease spinal loading in their workplaces.

## Highlights

- Low back pain (LBP) is linked to lifting style, posture, and spinal movement quality.
- Squat lifting lowers shear at lower spine but raises muscle effort.
- Stoop lifting increases shear loads, especially at L5/S1.
- Low back pain cases show reduced lumbar flexion, more pelvic compensation.
- Ergonomic design and training can lower LBP risk at work.

## INTRODUCTION

Low back pain (LBP) is one of the most prevalent disabling conditions among musculoskeletal disorders [1]. Most nations report a high lifetime prevalence rate of LBP, and this overall prevalence continues to rise largely secondary to a growing aging population [2]. The high occurrence and significant impact of LBP result in substantial socioeconomic expenses for workers, employers, and health care systems. Work-related physical loading on the lumbar spine stands as a primary occupational contributor to LBP development and remains one of the most reliable risk factors for modification [3].

The lumbar region of the spine is that part of the vertebral column between the thoracic spine and the sacral spine and is a common area of injury or pain [4]. The lumbar spine supports the upper body and facilitates trunk movement and load transfer during motion [5]. Manual handling work (lifting) and other biomechanical exposures in various occupations may result in injury or pain to the lumbar region [6]. Common lumbar spine biomechanical exposures in occupations include repeated lifting, prolonged awkward postures, trunk loading in non-neutral spinal positions, and non-neutral spinal movements [7,8]. Occupations with common lumbar spine biomechanical exposures include material handling jobs, logistics, manufacturing, healthcare, and transportation [8].

Lifting while at work has been the subject of extensive biomechanics and ergonomics research [9–11]. Lifting activities that involve a flexed trunk position or asymmetric posture without proper knee flexion generate compressive and shear forces on the lumbar spine, which may surpass tissue tolerance thresholds [12,13]. The risk associated with lifting tasks increases when repetitive lifting occurs or when workers perform tasks in a state of fatigue [14]. Although lifting with a neutral spine and symmetric posture while keeping weights close to the body's center of gravity reduces spinal load, these practices often remain impractical in work environments due to time constraints and inadequate employee training [15].

The lumbar spine is also affected by mechanical stress resulting from poor posture [16]. Remaining in one posture – such as sitting, standing, or stooping – for extended periods without movement or support can lead to fatigue of the supporting muscles, joint compression, and spinal misalignment [17]. Prolonged lumbar spine flexion increases disc pressure while simultaneously decreasing muscle activity. Standing likewise imposes a long-duration static load [18]. Encouraging movement variability may help reduce strain, although the optimal balance remains unclear [19].

Current studies on lumbar spine biomechanics in occupational environments demonstrate complex interactions between work-specific movements and the onset of LBP, which are influenced by numerous factors. The diversity of research methods and biomechanical modeling approaches creates challenges for comparing findings across studies. Although systematic reviews have examined specific aspects of this topic – such as the influence of lifting techniques or sitting posture on spinal loading or pain occurrence [20,21] – there remains a lack of comprehensive investigations exploring how combined lifting and movement patterns together with posture affect lumbar spine biomechanics and LBP risk among workers. This gap is important because occupational tasks typically involve complex, combined movements rather than isolated actions, making it difficult to translate existing evidence into practical and effective ergonomic recommendations. The authors hypothesized that the direction of lumbar spine movement (flexion/extension/rotation) is a modifiable exposure factor influencing LBP risk.

Therefore, this systematic review aimed to evaluate and synthesize current evidence on work-related movements, with a particular focus on lifting and posture, in relation to lumbar spine movement direction and the risk of LBP. The review also included investigations of movement characteristics, spinal loading, spinal alignment, and pain-related outcomes. Key methodological trends and commonly reported biomechanical outcome measures were identified and summarized. By clarifying these relationships, this review seeks to support evidence-based recommendations for workplace design, employee training, and injury prevention strategies to reduce the burden of LBP and promote musculoskeletal health in occupational settings.

## METHODS

### Search strategy

The authors conducted systematic literature searches in PubMed/Medline, Web of Science, and Scopus for publications from January 2015 until June 2025. The search parameters combined controlled vocabulary such as medical subject headings (MeSH) terms with free-text keywords that focused on lumbar spine or LBP as well as lifting and posture combined with occupational exposure and biomechanical loading. A systematic search strategy was developed based on the population, intervention/exposure, comparator, outcome (PICO) framework to ensure comprehensive coverage of relevant literature (Table 1). Additional literature was identified from citation chaining and by screening of reference lists of relevant reviews and included articles. Furthermore, Embase and CINAHL were not searched due to >90% overlap with included databases, though this is a potential limitation.

**Table 1.** Population, intervention/exposure, comparator, outcome (PICO) framework for search strategy of papers related to lumbar spine movement direction, spinal loading, and low back pain risk, January 2015 – June 2025

PICO element	Description	Keywords/MeSH terms
Population (P)	adult workers (≥18 years) involved in occupational tasks, especially in sectors like manufacturing, logistics, healthcare, transportation	“workers” OR “occupational groups” OR “ manual labor” OR “industrial workers” OR “nursing staff” OR “blue-collar” OR “occupational exposure”
Intervention/Exposure (I)	work-related physical activities: lifting, posture (static or dynamic), trunk movements, load transfer, repetitive motion	“lifting” OR “manual handling” OR “occupational lifting” OR “posture” OR “awkward posture” OR “stooping” OR “bending” OR “trunk movement” OR “repetitive motion”
Comparator (C)	not always applicable; studies may compare different postures or lifting styles, or exposed vs. non-exposed workers	“no lifting” OR “neutral posture” OR “symmetrical lifting” OR “sedentary work” OR “unexposed” OR “control group”
Outcome (O)	lumbar spine alignment/direction, biomechanical loading (e.g., disc pressure, shear/compression force), and/or low back pain risk, prevalence, or severity	“lumbar spine” OR “spinal loading” OR “biomechanics” OR “disc pressure” OR “low back pain” OR “lumbar strain” OR “spinal alignment” OR “musculoskeletal disorder”

### Study selection

All duplicate records were deleted before 2 independent reviewers screened the titles and abstracts of the remaining records. The same 2 reviewers accessed full texts from possible eligible studies for assessment against inclusion criteria. Disagreements were resolved by discussion with a third reviewer. The study selection process was conducted according to the PRISMA guidelines and summarized in a flow diagram.

### Inclusion and exclusion criteria

Studies were required to meet all of the following criteria:

– population: adult workers (≥18 years) in an occupational setting performing work activities including lifting, manual handling, prolonged standing, or work in non-neutral postures. Laboratory simulations of occupational tasks were also included, even if participants were not currently employed in those occupations;
– exposure: work-related biomechanical exposures affecting the lumbar spine, including lifting tasks, postural patterns (flexed, twisted, static), or combinations of movements in an occupational context;
– outcome: at least one of the following – quantitative measures of biomechanical load (e.g., compressive or shear forces) or LBP risk/severity. Studies that reported direction of lumbar spine movement (e.g., flexion, extension) were included only if this direction was analyzed as an exposure factor influencing biomechanical outcomes or LBP;
– study design: observational research (cross-sectional/cohort), experimental laboratory studies, or biomechanical modeling approaches;
– other: published in January 2015 – June 2025 in English; conducted in work-related environments or workplace simulations. The authors chose this time frame to reflect recent papers because biomechanical measurement technologies (e.g., motion capture, wearable sensors, EMG) have improved greatly, enhancing the accuracy of load estimates of the lumbar spine and direction of movement. The fundamental biomechanics of LBP remain unchanged, but measurement capabilities have substantially improved.

Studies were excluded if they met any of the following criteria:

– focused on non-occupational physical activity (e.g., sports movements or non-work-related lifting tasks);
– investigated spinal regions other than the lumbar spine without reporting lumbar-specific outcomes (e.g., thoracic or cervical regions);
– case reports, editorials/commentaries, conference abstracts, narrative reviews, or articles that did not report original data;
– used *in vitro* or animal models without incorporating human-specific biomechanical data;
– did not quantitatively or qualitatively assess spinal movement, spinal loading, or LBP outcomes.

### Data extraction

Two reviewers independently extracted data using a standardized form. Study characteristics (authorship, publication year, origin country, research design, participant count, occupation category, age range of participants, and movement type investigated) and outcome data (lumbar spine alignment measurement results or directional data with biomechanical loading metrics and LBP outcomes) were extracted. Measurement tools (e.g., motion capture, electromyography [EMG], modeling, or questionnaires) were also recorded. Disagreements were resolved through discussion or a third reviewer. Data were compiled into 2 summary tables: 1 for general study characteristics and 1 for key exposures, outcomes, and findings.

### Risk of bias assessment

The methodological quality of the studies was assessed, and risk of bias (ROB) was evaluated using the Risk Of Bias In Non-randomized Studies – of Interventions (ROBINS-I) tool [22,23]. Five domains were rated: bias due to confounding, selection of participants, missing data, deviations from intended interventions, and measurement of outcome. The ROB for each domain was rated as low, moderate, serious or critical. The overall ROB was then derived from the most serious ROB for each domain. Two reviewers independently performed the assessment and disagreements were solved by consensus.

### Data synthesis

Due to heterogeneity in study design, exposure definitions, biomechanical measurement methods, and outcome metrics, a narrative synthesis approach was adopted. Findings were grouped and compared based on:

– type of biomechanical exposure (e.g., lifting, static posture, dynamic tasks);
– measured lumbar spine movement (e.g., flexion/extension);
– outcome domain (e.g., loading forces, alignment, or pain incidence).

## RESULTS

### Study selection

A total of 738 articles were identified through the database search. After the removal of 527 duplicates, 211 records remained for screening. Based on their titles and abstracts, 193 studies were excluded because they were either irrelevant or did not meet the eligibility criteria. All 18 full-texts were screened and assessed for eligibility and 8 studies were excluded for the following reasons: inadequate outcome measures (N = 3), non-human or *in vitro* studies (N = 5). Finally, 10 studies were included in the final synthesis (Figure 1).

**Figure 1.**
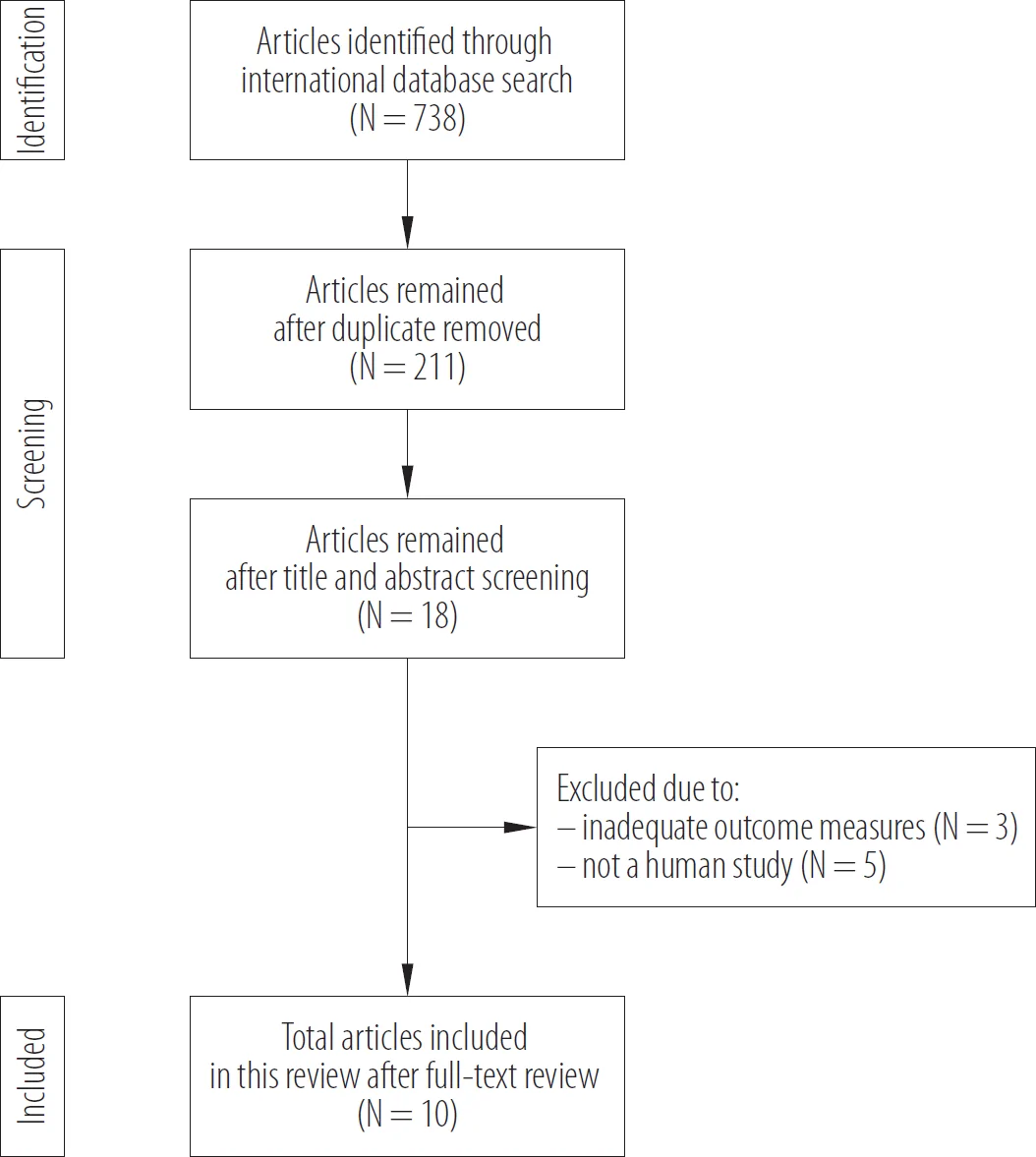
Flowchart of eligible studies included in the systematic review related to lumbar spine movement direction, spinal loading, and low back pain risk, January 2015 – June 2025

### Study characteristics

The 10 studies were published in 2017–2025 [24–33]. The studies originated from various countries, including France [24], the United States [25,27,28,33], Belgium [26], Iran [29], Switzerland [30], Brazil [31], and Malaysia [32]. The research incorporated experimental laboratory-based studies, modeling/simulation, and observational cross-sectional studies. Most studies focused on healthy adult participants [26–33], with some comparing individuals with and without LBP [24,25]. The sample sizes ranged from 7 to 204 participants. The studied occupational tasks included lifting in various styles (e.g., squat, stoop, one-handed), posture-related exposures (e.g., confined lifting, prolonged standing, asymmetry), and dynamic tasks such as walking or load transfer. Tasks were measured in an occupational workplace as well as a simulated occupational environment in the laboratory. A summary of the study characteristics including the country of origin, sample population, age, movement task and design are shown in Table 2.

**Table 2.** Summary of study characteristics related to lumbar spine movement direction, spinal loading, and low back pain risk (LBP), January 2015 – June 2025,

Study	Year	Country	Study design	Study setting	Sample size [n]	Population/Occupation	Age (M±SD)	Type of movement/Task investigated
Courbalay et al., 2017 [24]	2017	France	experimental pilot study	laboratory	LBP: 17, control: 18	adults (with and without LBP)	with LBP: 34.2±14.08 years without LBP: 32.5±11.75 years	unconfined lifting and lowering with varied load expectations
Shojaei et al., 2017 [25]	2017	USA	experimental	laboratory	LBP: 19, control: 19	female adults (with and without acute LBP)	n.r.	trunk forward bending and backward return
Have et al., 2019 [26]	2019	Belgium	experimental	laboratory	10	healthy adults	31.5±12.7 years	unconfined lifting
Weston et al., 2020 [27]	2020	USA	experimental	laboratory	10	baggage handlers	25.2±6.4 years	lifting in confined vs. unconfined spaces
Weston et al., 2020 [28]	2020	USA	experimental	laboratory	30	healthy adults	male: 27.0±6.4 years, female: 26.9±3.9 years	1-handed vs. 2-handed lifting
Khoddam-Khorasani et al., 2020 [29]	2020	Iran	simulation/modeling study	laboratory	15	healthy adults	30±6 years	lifting in lordotic vs. kyphotic lumbar posture
von Arx et al., 2021 [30]	2021	Switzerland	experimental	laboratory	30	healthy adults	31.8±8.5 years	freestyle vs. squat vs. stoop lifting
Gomes et al., 2023 [31]	2023	Brazil	cross-sectional	warehouse/field	204	warehouse workers	31.0±6.7 years	manual handling, stock sorting
Rahman et al., 2025 [32]	2025	Malaysia	cross-sectional	hospital/field	LBP: 13, control: 13	nurses	n.r.	walking and 180° load transfer
Refai et al., 2025 [33]	2025	USA	experimental	laboratory	7	healthy adults	31.7±7.0 years	lifting at different NIOSH difficulty levels

n.r. – not reported.

NIOSH – National Institute for Occupational Safety and Health.

### Key data and outcome measures of included studies

All included studies assessed at least one of the following exposure or outcome measures: exposure – direction of lumbar spine movement (e.g., flexion, extension, rotation) or postural alignment; outcomes – spinal loading metrics (e.g., compressive/shear forces) or LBP risk/severity (Table 3).

**Table 3.** Summary of key data and outcome measures related to lumbar spine movement direction, spinal loading, and low back pain risk (LBP), January 2015 – June 2025

Spine segment assessed	Exposure type	Exposure duration/load	Measurement tool	Lumbar spine movement	Outcome		Key finding	Ref.
					biomechanical	pain-related		
L1 to S1	freestyle lifting (varying load expectation)	5 lb, 12.5 lb, 20 lb	EMG (erector spinae, vastus lateralis), kinematics	not significant in lifting phase; angles measured	EMG activity modulated by expectation and pain group	self-reported exertion, pain history, functional disability	LBP group had reduced vastus lateralis activity during the lifting/lowering of the heaviest (20 lb) weight; no significant kinematic differences between groups	24
L1 to S1	trunk bending task	multiple paced trials, no external load	motion capture (kinematics)	lumbar flexion, pelvic and thoracic rotation	angular velocity, acceleration, deceleration of lumbar flexion	acute LBP (diagnosed ≤3 months)	acute LBP patients: ↓ lumbar flexion and velocity, ↑ pelvic motion; group × age × pace interactions were significant	25
L1 to S1	lifting (stoop vs. squat)	repeated lifts with 10 kg box	motion capture and peak EMG	flexion and extension	peak joint	n.a.	squat lifting increased total musculoskeletal effort, especially in limbs; lumbar spine loading similar in both techniques	26
L1 to S1	confined vs. unconfined lifting	14.5–22.7 kg bags, 2 lift reps	EMG-driven model	flexion	compression and shear	n.a.	high compressive loads in both confined and unconfined lifting; kneeling slightly reduced shear	27
L1 to S1	1- vs. 2-handed lifting	2.7 kg, 7.6 kg, 11.3 kg	EMG-driven model and motion capture	trunk rotation and flexion	compression and shear	n.a.	one-handed lifts increased lateral shear but reduced compression compared to 2-handed lifting	28
T12 to S1	postural variation (lordotic vs. kyphotic)	simulated lifts	EMG + FE + MS model	lumbar curvature and trunk forward flexion	segmental compression/shear	n.a.	lordotic (arched) lumbar posture during lifting increased segmental L1–S1 compression (+7 to 46%) and shear (+5 to 48%) forces, relative to kyphotic posture	29
L1 to S1	lifting style comparison	15 kg box	whole body motion capture	lumbar flexion	compressive and shear loads	n.a.	stooping ↓ compression but ↑ shear (except L5/S1)	30
L1 to S1	manual handling tasks	self-reported exposure	questionnaire and strength test	n.a.	n.a.	LBP prevalence (24%)	separator tasks ↑ LBP risk; higher strength ↓ LBP prevalence	31
L1 to S1	walking with load transfer	standard load during walking	motion capture	lumbar flexion, pelvic tilt	n.a.	self-reported exertion, pain history	LBP nurses: ↑ lumbar flexion and pelvic tilt during tasks	32
L5/S1	NIOSH lifting levels	20 and 21 kg	EMG-driven model	trunk rotation	L5/S1 compression and shear	n.a.	model-estimated loads exceeded NIOSH thresholds consistently	33

n.a. – not applicable.

EMG – electromyography; FE – finite element; L1 – first lumbar vertebra; L5 – fifth lumbar vertebra; MS – musculoskeletal; S1 – first sacral vertebra; T12 – twelfth thoracic vertebra.

NIOSH – National Institute for Occupational Safety and Health.

Findings were stratified by study design to reflect differing levels of evidence. Of the 10 studies, 7 were experimental laboratory studies [24–28,30,33], 1 was a biomechanical modeling study [29], and 2 were observational field studies (cross-sectional) [31,32]. Refai et al. [33] was classified as an experimental laboratory study because it involved human participants performing lifting tasks in a controlled laboratory environment, with EMG-driven biomechanical modeling used to estimate spinal loading outcomes. Experimental laboratory studies consistently demonstrated that stoop lifting increases shear forces at L5/S1, while squat lifting reduces shear but increases overall musculoskeletal effort. Biomechanical modeling studies reported segmental compressive and shear forces during different lumbar curvatures, showing lordosis increases spinal forces. Observational field studies found associations between manual handling activities and increased LBP incidence, but cannot confirm causality.

Investigations into lifting techniques have typically compared squat, stoop, freestyle, and asymmetrical lifting styles. For instance, Weston et al. [27] and von Arx et al. [30] both reported that, while squat lifting resulted in greater overall musculoskeletal loading, stoop lifting produced higher shear forces at specific spinal levels. Khoddam-Khorasani et al. [29] quantified spinal loading using finite element modeling combined with EMG under neutral and varying lumbar curvatures, demonstrating that a lordotic posture resulted in greater compressive and shear loads. Neuromechanical data from individuals with chronic or acute LBP (e.g., Courbalay et al. [24], Shojaei et al. [25]) have revealed differences in muscle activation patterns, reduced lower lumbar flexion velocity, and compensatory pelvic kinematics during lifting or bending tasks. Field-based cross-sectional studies (Gomes et al. [31], Rahman et al. [32]) employed questionnaire data and motion analysis to show that frequent manual handling, reduced physical strength, and specific occupational roles were associated with higher LBP prevalence and altered trunk mechanics. Researchers commonly utilized 3-dimensional motion capture systems in combination with EMG and OpenSim (Stanford University, Stanford, USA) musculoskeletal modeling with finite element analysis. Laboratory-based studies predominantly reported peak compressive forces at the L5/S1 level ranging from moderate (approx. 1500 N) to high (>3000 N), with substantial variation depending on lifting technique and load magnitude.

### Risk of bias assessment

Risk of bias was evaluated for all included studies using ROBINS-I tool (Table 4). Three studies: Shojaei et al. [25], von Arx et al. [30], and Refai et al. [33], were considered to be at low ROB overall, as all were experimental in nature, conducted in a laboratory environment, and applied a clear, well-defined biomechanical exposure protocol. The study by Khoddam-Khorasani et al. [29] was considered moderate ROB due to the possibility of limitations related to exposure classification and assumptions used in the model development, as no human participants were actually exposed to the *in situ* loading conditions of interest. The cross-sectional study by Rahman et al. [32] was considered moderate ROB, as this study design is more susceptible to confounding and limited control over measurement of exposure. The survey-based observational study by Gomes et al. [31] was considered to be at serious ROB, as it was reliant on self-report of exposure and outcome data, which may have led to increased recall and reporting bias. Overall, laboratory-based experimental studies provided the strongest internal validity, with more precise definitions of exposures, while observational studies and simulation studies had a higher susceptibility to these methodological limitations. Figure 2 presents the distribution of ROB domains across all included studies.

**Table 4.** Risk of bias (ROB) assessment in papers related to lumbar spine movement direction, spinal loading, and low back pain risk, January 2015 – June 2025

Study	Confounding	Selection of participants	Missing data	Deviations from intended interventions	Measurement of outcomes	Overall ROB
Courbalay et al., 2017 [24]	low	low	low	low	moderate	moderate
Shojaei et al., 2017 [25]	low	low	low	low	low	low
Have et al., 2019 [26]	low	low	low	low	moderate	moderate
Weston et al., 2020 [27]	low	low	low	low	moderate	moderate
Weston et al., 2020 [28]	low	low	low	low	moderate	moderate
Khoddam-Khorasani et al., 2020 [29]	moderate	low	low	n.a. (simulation)	low	moderate
von Arx et al., 2021 [30]	low	low	low	low	low	low
Gomes et al., 2023 [31]	moderate	low	low	moderate	serious	serious
Rahman et al., 2025 [32]	moderate	low	low	low	low	moderate
Refai et al., 2025 [33]	low	low	low	low	low	low

n.a. – not applicable.

**Figure 2.**
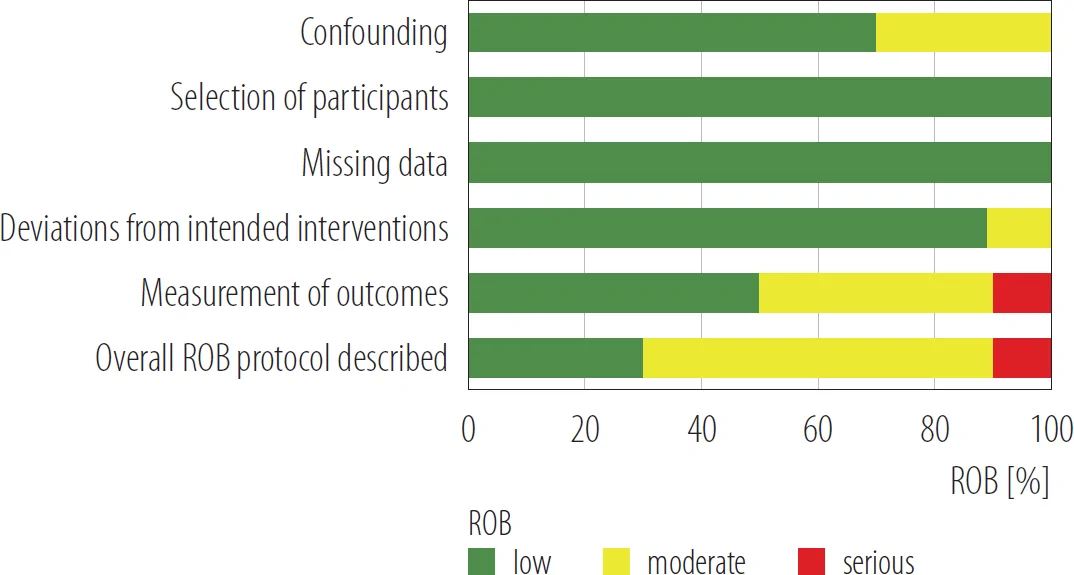
Distribution of risk of bias (ROB) domains across all included studies related to lumbar spine movement direction, spinal loading, and low back pain risk, January 2015 – June 2025

### Study-level synthesis of included studies

Among the experimental laboratory-based studies, Weston et al. [27] compared squat and stoop lifting and found that squat lifting increased total musculoskeletal loading, while stoop lifting increased shear loading at the L5/S1 level. Likewise, von Arx et al. [30] found increased spinal shear forces during stoop lifting, although lower compressive forces compared to squat lifting, highlighting a trade-off between different spinal loading patterns. Courbalay et al. [24] and Shojaei et al. [25] examined the biomechanics of individuals with LBP and reported modified neuromuscular strategies, such as decreased lumbar flexion speed and increased pelvic tilt, which indicated potential adaptive or compensatory movements when lifting. Refai et al. [33] examined spinal loading under constrained lifting conditions and found that limited motion resulted in increased local spinal loading.

The biomechanical modeling study by Khoddam-Khorasani et al. [29] provided further insight into internal spinal loading mechanisms. They used a finite element model and EMG-driven boundary conditions to measure compressive and shear forces under different lumbar curvatures and found that a lordotic posture led to higher compressive and shear forces. These findings were consistent with simulation-based analyses that emphasized the sensitivity of spinal loads to posture and alignment.

Observational studies offered complementary real-world perspectives. Gomes et al. [31], based on survey and motion analysis, observed manual handling and occupational demands were linked to increased risk of LBP but ROB increased due to reliance on self-reports. Rahman et al. [32] conducted a cross-sectional study linking reduced physical capacity and repetitive lifting exposure with altered trunk kinematics and increased LBP risk, although causal relationships could not be established due to study design limitations.

### Cross-study synthesis of evidence

Across the ten included studies, 3 consistent patterns emerged despite heterogeneity in design. First, studies on lifting technique (6 studies [24,25,27,29,30,33]) found stoop lifting consistently led to greater L5/S1 shear forces than squat, with squat lifting involving higher total musculoskeletal load (a trade-off likely to influence injury risk over time). Second, regarding spinal posture [29,33]), lordotic and constrained postures increased segmental compressive and shear forces compared to neutral posture. Third, observational studies [31,32] reported higher LBP prevalence among workers performing repetitive manual handling or with reduced physical capacity, aligning with the biomechanical findings. No study found that neutral or symmetric postures increased LBP. The uniformity of this directional effect suggests the direction of movement is a modifiable exposure factor.

Overall, while experimental and modeling studies provided high-resolution biomechanical data under controlled conditions, observational studies contributed broader contextual evidence regarding occupational exposure and LBP prevalence. The consistency of these findings across study designs supports the evidence that lifting technique, spinal posture and direction of movement play critical roles in determining spinal load and LBP risk, despite the different designs and measurement strategies used.

### Interpretation of main findings

The findings of this review highlight that both lifting mechanics and static or dynamic postures in occupational tasks significantly influence lumbar spine loading and the direction of spinal movement. Several studies [27,30] found that while squat lifting reduces shear forces at lower vertebral levels, it may increase total musculoskeletal effort elsewhere, indicating a biomechanical trade-off. In contrast, stoop lifting consistently produced greater shear loads, especially at the L5/S1 segment, suggesting a higher risk of injury despite lower energy demands.

Task specific studies with LBP subjects [24,25] reveal altered neuromechanical strategies such as less lumbar flexion, lower angular velocity, and increased pelvic compensation to trunk movement. While this may be an avoidance of painful movement, it may create secondary strain and chronic dysfunction if these adaptations are long term [34,35]. Notably, posture played a critical role independent of lifting. The modeling study by Khoddam-Khorasani et al. [29] revealed significantly increased compressive and shear forces during lifting with a lordotic posture, emphasizing the importance of spinal curvature during movement. Field studies [e.g., 31,32] showed a strong association between job roles involving repetitive manual handling or constrained movements and higher LBP prevalence.

Cumulatively, these data support the authors' *a priori* hypothesis (stated in the introduction) that the direction of spinal motion, specifically flexion and rotation of the spine while lifting a load, is a critical factor influencing lumbar strain and risk of LBP. The existence of asymmetrical movement, restricted lifting, and insufficient strength also add to the strain during lifting. Research shows that lifting with a neutral spine reduces injury risk but occupational settings rarely allow such methods because workers lack ergonomic training.

It is important to consider that LBP is not solely an occupational disorder but a complex and multifactorial condition. Evidence from large-scale epidemiological studies, including the Lancet series on LBP [35], demonstrates that the vast majority of cases are classified as non-specific, with no clearly identifiable structural or pathological cause. Rather, LBP is affected by a broad array of non-work factors including lifestyle (e.g., sedentary lifestyle, smoking) and psychological (distress, perceptions of pain) factors, as well as other medical conditions, and socioeconomic status. In particular, people with low socioeconomic status and with psychological distress are at greater risk of persistent and disabling LBP. Also, it tends to recur and the role of central pain modulation processes is emerging as particularly important in chronicity. In this regard, although the current review notes the biomechanical effects of occupational lifting and posture on lumbar biomechanics, these should be considered within a biopsychosocial context. Ignoring non-occupational factors may result in a bias towards overestimating the contribution of occupational factors to LBP, which can result from the interaction of multiple individual, social and occupational factors.

### Preclinical and review evidence

Experimental and modeling research has shown that the spine can withstand substantial loads under specific conditions [36]. For example, force diagrams of acrobatic jumps performed by circus artists have shown peak shoulder forces of approx. 1700–1800 N during the launch phases – far exceeding typical occupational loads [37]. This illustrates that while daily work tasks impose lower absolute forces, the underlying biomechanics – namely the combination of compressive and shear loading – can be quite similar.

Cadaveric and *in vitro* studies have further confirmed that flexion under compressive loading significantly increases the risk of injury to intervertebral discs and ligaments. Dolan et al. [38] found that stoop lifting reduces muscular torque by about 10% but increases spinal bending moments by up to 75%. Moreover, increases in load weight, size, or distance from the body lead to significant rises in both compressive and bending moments. These findings emphasize that excluding bending forces from risk assessments provides an incomplete picture of lumbar injury mechanisms. Notably, the findings of von Arx et al. [30], which demonstrated lower compressive and total lumbar loads during stoop lifting than during squat lifting, but generally higher shear loads at the upper lumbar segments, align with biomechanical evidence from non-occupational contexts [39]. Importantly, at the L5/S1 segment, anterior shear loads were lower during stoop lifting than during squat lifting [30]. These findings reinforce the segment-specific nature of lumbar loading and indicate that the biomechanical effects of lifting technique depend on the spinal level and loading component considered.

Other related review articles also offer complementary insights. A systematic review by Jahn et al. [40] found moderate evidence linking heavy lifting and non-neutral trunk postures to chronic LBP, though their inclusion of both occupational and recreational lifting limited generalizability. Swain et al. [41], in an umbrella review of 41 systematic reviews, reported conflicting findings regarding the association between spinal posture and LBP, concluding that causality remains uncertain. Similarly, Saraceni et al. [42] noted no consistent difference in lumbar flexion angles between individuals with and without LBP during lifting, raising questions about the predictive power of motion patterns alone. In contrast, the authors' review revealed that participants with LBP exhibited consistently altered neuromechanical behaviors – such as reduced lumbar flexion velocity and increased pelvic compensation (e.g., Shojaei et al. [25], Rahman et al. [32]) – which were less emphasized in prior meta-analyses. This difference likely stems from 2 factors: first, previous meta-analyses (e.g., Saraceni et al. [42]) primarily pooled cross-sectional studies that measured only peak lumbar flexion angles, which may not capture dynamic movement patterns like velocity; second, prior reviews often included both occupational and recreational lifting in general populations, whereas the authors' review focused exclusively on occupational or simulated workplace tasks where fatigue and repetition may amplify neuromechanical adaptations. Moreover, the inclusion of studies employing advanced load-estimation methods – including an experimental laboratory study that incorporated an EMG-driven musculoskeletal model [33] and a dedicated finite element modeling study [29] – allowed a segment-specific examination of compression and shear forces under realistic workplace scenarios, providing a level of mechanistic granularity often lacking in earlier reviews. The systematic reviews (e.g., Jahn et al. [40], Swain et al. [41]) primarily used epidemiologic or observational data without biomechanical modeling, which cannot estimate spinal forces or segmental spinal loading. Inclusion of studies that incorporated biomechanical modeling techniques, together with experimental laboratory investigations, may explain why the authors' review identified directional and segmental effects that were not emphasized in previous reviews.

In conclusion, while supporting preclinical and review data demonstrate that posture and lifting style are biomechanically relevant to load and injury risk, to the best of the authors' knowledge, this is the first systematic review to consider spine movement direction and applied load, as well as to be clearly linked to real-life occupational tasks. In this way, the authors' results differ from previous reviews, which instead considered overall LBP prevalence or nonspecific ergonomic guidelines. The authors have identified lifting strategies and spine mechanics during lifting with clear directions, which are both easily modifiable and specific to the occupational setting, making the results both more interpretable and actionable for workplace intervention.

### Limitations

This review was subject to a number of limitations, which are listed below:

– Due to substantial heterogeneity in study designs, exposure definitions, outcome measures (e.g., compression forces, shear forces, LBP prevalence), and measurement tools (e.g., EMG, motion capture, modeling), a meta-analysis was not feasible. A quantitative synthesis would have provided more precise effect size estimates and a relatively definitive conclusion, which the current narrative synthesis cannot offer.
– Laboratory studies provide a controlled environment, but lack ecological validity, whereas field studies are more generalizable but may be subject to self-reported outcomes and less control over variables.
– Sample sizes were often small in many studies, while several investigated only healthy populations, limiting generalizability to other working populations, including older adults and those with chronic LBP.
– Simulation studies were useful in understanding the implications of various exposures, but they were limited by the modeling assumptions that are often different from human biomechanics.
– Methods of measurement were diverse – from EMG and motion capture to biomechanical modelling – limiting direct comparisons.
– Some studies had moderate to serious ROB, specifically those with observational designs or self-reported data.

### Recommendations for future research

Future studies should prioritize large-scale, longitudinal designs to assess cumulative effects of occupational lifting and posture over time. The use of wearable sensors could improve data accuracy in real-world settings. Research should also evaluate ergonomic interventions tailored to high-risk occupations, measuring both biomechanical impact and practical feasibility. Moreover, further work is required to understand movement variability and fatigue interactions with spinal loading and injury. Finally, future research needs to adopt interdisciplinary methods that merge biomechanical data and psychosocial and organizational movement behavior factors for better LBP prevention at work.

## CONCLUSIONS

This systematic review reveals that lumbar spine movement direction and type (as exposure factors) during lifting activities and sustained postures significantly influence spinal load and LBP risk for workers. All ten studies (experimental, modeling, and observational) consistently demonstrate that stooped, asymmetrical, lordotic and constrained postures increase segmental spinal loading (especially shear forces at L5/S1) and are associated with altered neuromechanical responses or increased LBP prevalence. None of the studies found a beneficial effect of non-neutral postures. Although a meta-analysis was not possible due to heterogeneity, consistency of directional evidence from various study designs allows the authors to conclude that direction of movement is a potentially modifiable factor. Lumbar spine load at L5/S1 was significantly elevated for stooped postures, and asymmetrical and constrained lifting movements.

Additionally, individuals with LBP exhibited altered compensatory movement patterns, suggesting both biomechanical and neuromuscular adaptations to pain. Despite reducing injury risk through squat lifting and neutral spinal alignment these methods face practical implementation difficulties in workplace environments. Safe movement behaviors must be promoted through ergonomic training combined with both task redesign and supportive technological solutions. The findings from this review will guide the development of occupational interventions which modify movement patterns to reduce injury risk. The combination of biomechanical literacy programs with real-time monitoring systems and improved lifting techniques enhances work-related LBP prevention and management. These findings should be interpreted within the broader biopsychosocial framework of LBP, where occupational biomechanical exposures interact with lifestyle, psychological, and socioeconomic determinants.

## AI USE

This paper was written with the assistance of GPT-5 to enhance the grammatical accuracy and fluency of the content, ensuring that the final text meets high standards of clarity and coherence. The use of GPT-5 has been integral in refining the language, while the intellectual contributions of the authors remain central to the research and conclusions presented.
